# Challenges in Personalized Nutrition and Health

**DOI:** 10.3389/fnut.2018.00117

**Published:** 2018-11-29

**Authors:** Meghna Verma, Raquel Hontecillas, Nuria Tubau-Juni, Vida Abedi, Josep Bassaganya-Riera

**Affiliations:** ^1^Nutritional Immunology and Molecular Medicine Laboratory, Biocomplexity Institute of Virginia Tech, Blacksburg, VA, United States; ^2^Graduate Program in Translational Biology, Medicine and Health, Virginia Tech, Blacksburg, VA, United States; ^3^Department of Biomedical and Translational Informatics, Geisinger Health System, Danville, PA, United States

**Keywords:** personalized nutrition, health, machine learning, artificial intelligence, data analytics, electronic health record, infrastructure

## Introduction

### Personalized nutrition and approaches employed

Personalized nutrition refers to tailored nutritional recommendations aimed at the promotion, maintenance of health and prevention against diseases (1). These recommendations take into account differential responses to certain individualized food-derived nutrients that arise due to the interaction between nutrients and biological processes (2). These include the interactions between internal factors such as genetics, microbiome, metabolome interactions as well as external factors such as dietary habits and physical activity (3). In contrast to precision medicine defined by the Precision Medicine Initiative (https://obamawhitehouse.archives.gov/node/333101) as an approach toward the treatment and prevention of disease for an individual, the goal of personalized nutrition is to promote the health and well-being through diet.

A balanced diet promotes good health as it provides adequate amounts of energy, proteins, vitamins, minerals, essential fats, micro, and macronutrients for the metabolic needs of the body to function properly at each stage of the lifespan. The absence of balanced food and nutrition security leads to health problems such as diabetes, obesity, and malnutrition (4). Although, the importance of nutrition and beneficial effects of food are well established, the mechanisms underlying their role in disease prevention or health benefits are incompletely understood (5, 6). Further, there exists an inter-individual response to dietary intervention due to which a sub population may benefit more than others. This underlying variability can be attributed to genetics, age, gender, lifestyle, environmental exposure, gut microbiome, epigenetics, metabolism nutrition derived from diet, and foods. The inter-individual variability to treatments and nutritional recommendations is largely reflected in biomarker values (7).

Reductionist approaches fail to demonstrate how the cellular and molecular responses due to food produce health benefits (6). Current approaches used to study the inter-individual response to diet include–omics technologies such as genomics, metabolomics, proteomics integrated with the systems biology programs. These approaches are focused on integrating and analyzing complex datasets generated during dietary intervention association studies (3, 8, 9). Systems biology approaches are impacting the field of nutrition (10–12) and immunology (13), however, significant challenges still remain in the translation and application of these advances to human studies (9). A comprehensive systems-wide mechanistic understanding of the interplay between nutrition and health benefits requires the knowledge of network dynamics in the context of health, pre-disease, and disease states. This requirement gives rise to the demand for new approaches and methods that could not only quantify the effects of dietary interventions in healthy individuals but also facilitate comparison to diseased patients (6).

### Need for integrated personalized predictive models for use in personalized nutrition and health

To understand the underlying health dynamics while considering inter-individual variability and implementing personalized nutrition-driven interventions, efforts should focus on devising predictive methods that timely monitor the individual's health responses to food. A systems science perspective can help physicians tailor targeted treatment, comprehend the variability in response to treatments and design personalized nutrition approaches (14). Personalized nutrition approaches have the potential to spearhead the creation of information-processing representations of digestion, absorption, and metabolism. These provide linkages between molecular events and health outcomes through: (i) integration of data at all salient scales; (ii) combination of mulsticale models with health outcomes through advanced machine learning (ML) models; (iii) generation of non-intuitive hypotheses; and (iv) experimental validation using preclinical and clinical trials with standardized nutritional interventions. With the advent of big-data era, data specific to consumption of standardized meal, functional food, and beverage sales reports can be extracted. Health informatics enabled initiatives can be applied to conduct data mining and extraction from the electronic health records (EHRs) and insurance claims database. The EHR data can be combined with knowledge derived from nutritional and data sciences to build computational models and synthetic patient cohorts. These synthetic patients can be used as avatars that reflect inter-individual variation to preform predictive analysis and evaluate the system-level responses to the personalized food recommendations. These predictive insights can be utilized in order to elucidate the complex regulatory mechanisms of nutritional interventions at the interface of immunity, metabolism, and gut microbiome. Overall, advanced computational methods and data analytics platforms could help shape the development of health platforms, tailor future nutritional recommendations for promoting health and accelerating the translation of the recommendations into the clinic.

Recent studies demonstrated successful application of providing personalized dietary advice at an individual level (15, 16). Although the prediction method used by Zeevi et al. demonstrated the effectiveness of personalized diet regimes to reduce levels of glucose, the results failed to connect to health outcomes. A web-based pan-European, Food4Me study (17) aimed to evaluate whether personalized advice caused more changes in dietary behavior as compared to “one size fits all” approach (16). An automated dietary feedback system was used to deliver personalized dietary advice and its comparison with manual system demonstrated complete agreement (17). The study demonstrated that personalized nutrition advice was more effective compared to a population-based nutritional advice.

**Figure 1:**
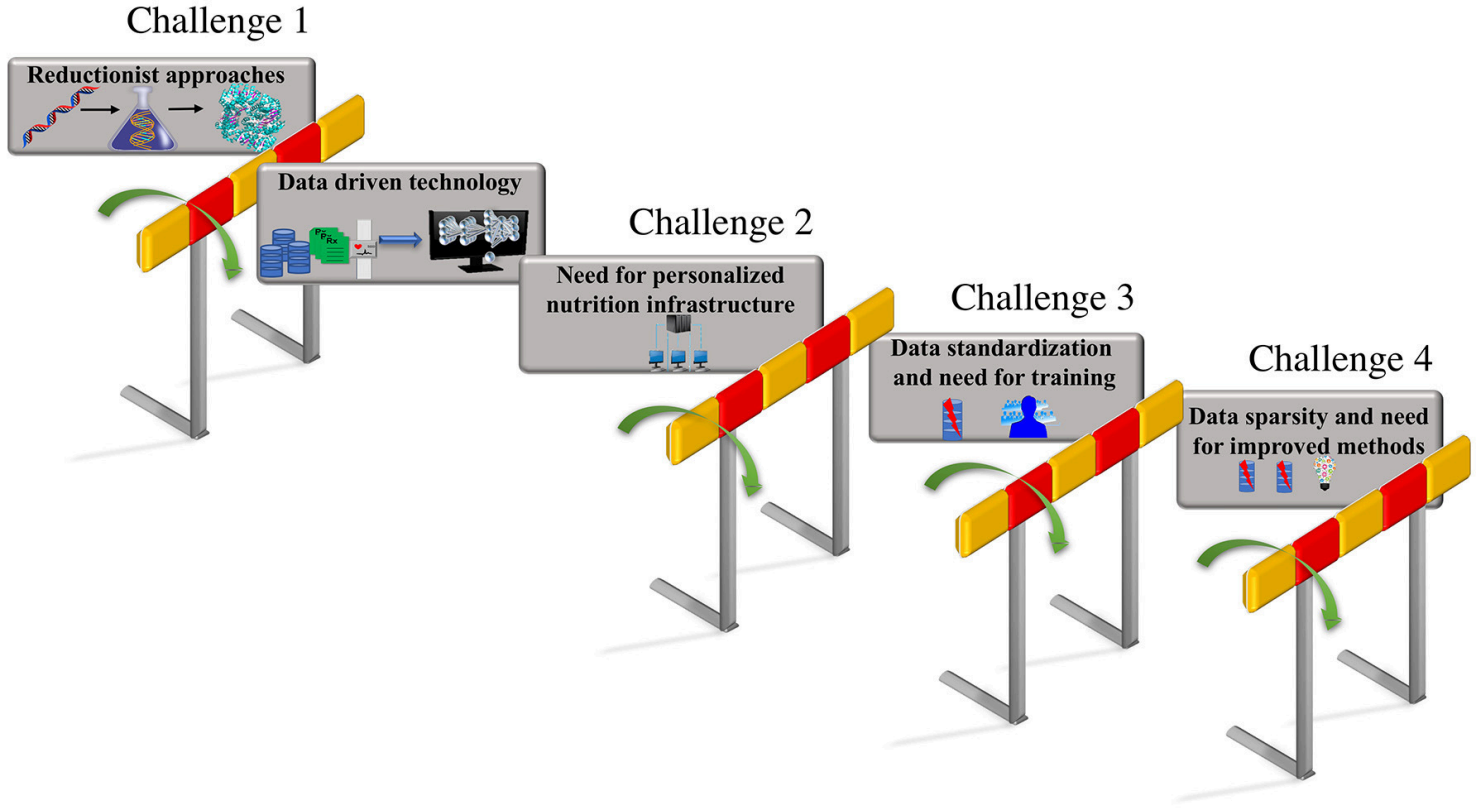
Challenges in personalized nutrition. The challenges encountered in the path of making tailored recommendations toward personalized nutrition and health include—(i) limitations due to the reductionist approaches that can be overcome by employing data-driven technologies such as AI and ML; adaptation to existing data-driven technologies raises, (ii) the need for building a personalized nutrition computational infrastructure; the lack of standardization in format of the data utilized in electronic health records raises, (iii) the need for data standardization and updated training programs for the users; the inconsistencies and missing values in the electronic health records results in, (iv) the data sparsity and missing data problem that emphasizes the need for the development of new methods for data imputation.

These integrated data-driven approaches that build predictive computational models could be trained with additional features including phenotypic changes due to nutritional factors and changes due to interaction between genotypic and nutrient derived metabolic factors. The predictions from these models that include genotypic and metabolic features can aid the design of personalized diets based on the feature attributes that capture human diversity and variation. Thus, a unique comprehensive strategy that can automate data driven analytical model building, could be employed by focusing on the unique iterative integration of large-scale clinical record mining, -omic analysis, hypothesis-based modeling, simulation, and advanced ML approaches, to make tangible progress toward personalized nutrition and precision medicine. The vision for personalized nutrition has stimulated an immense interest for advancements in the diagnostics and decision support systems that allow continuous assessments of nutritional status. However, the advancement in the predictive technologies and their integration has posed numerous challenges. The focus of this review article is to put forth the major challenges (as shown in Figure 1) encountered in the process of revolutionizing the personalized nutrition health-care information technology including (i) limitation in the reductionist approaches and opportunities for adoption of advanced computational data-driven technologies; (ii) need for personalized nutrition computational infrastructure; (iii) data standardization and the requirement for training individuals; (iv) data sparsity, missing data and need for improved imputation methods. We further discuss the possible solutions for above listed challenges to achieve preventive, personalized and predictive approaches that aid the process of making decisions about diet and foods at an individual personalized level.

## Challenges

### Challenge 1: limitation in reductionist approaches and opportunities for adoption of advanced computational data-driven technologies

The study of biological mechanisms at a single gene or protein level in nutritional studies is largely outdated. The dynamic interaction among nutrition-metabolism-microbiota-host at the cellular and molecular level and health outcomes at the individual and personalized level are not completely understood (5, 6). A holistic understanding of health requires evaluating the interactions among diet, genes, gene products, health, and environmental exposures as opposed to focusing on the interaction of nutrients or macromolecules on specific gene or gene products. Current experimental techniques are limited in their ability to—(i) allowing researchers to quantitatively manipulate diet in a controlled manner in humans and animals and (ii) being able to trace events at the tissue level back to specific cellular and molecular level interactions and or signaling mechanisms. Data-driven approaches that utilize multi-parameter measures such as the influence of the nutrients on gene expression, genetic variations and interaction with environmental factors such as influence of lifestyle measures on gut microbiome interaction are capable of providing a comprehensive understanding of an individual's health.

The current health care system evaluates static measurements of an individual's health which includes the EHR data contents and physical examination results. In addition to the static measurements continuous measurements of health determinants such as the daily meal intake, microbiota composition, metabolomes, sleep, and stress levels can allow for stratification of patients in sub-groups. An establishment of a baseline range for every parameter of a healthy individual is crucial (18). The above listed multi-parameter measures will not only capture the dynamic relationship between the healthy parameter range of an individual but also aid in the process of early detection of the diseases and provide support in the decision-making processes. Advances in computational modeling and tools, data analytics methods, and a systems science approach (4) can be employed to design, update strategies for nutrition-based health care, and enhance disease preventive management.

#### The use of electronic health records, data from wearables, and health apps integrated with individual specific variables—a “big data” approach

**Figure 2:**
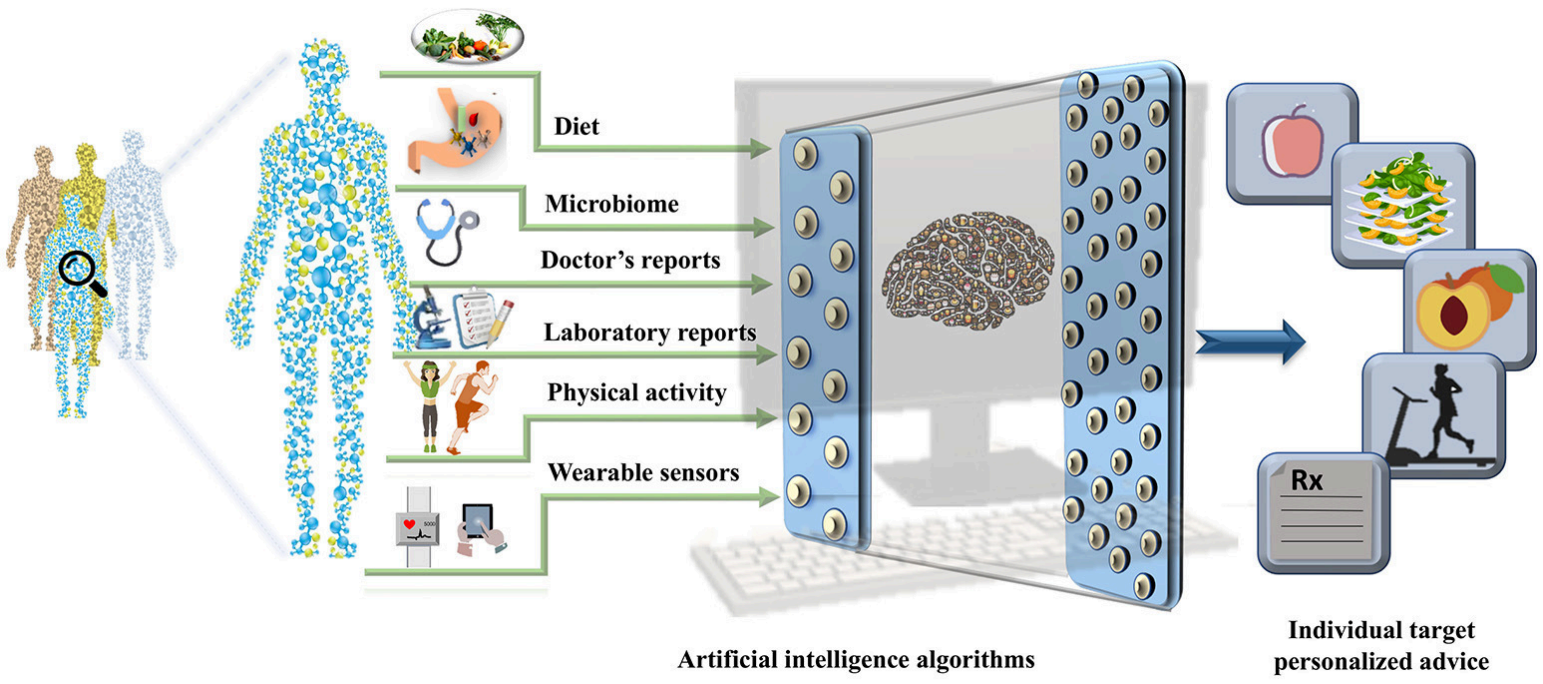
A pipeline for personalized nutrition and health. The figure represents the integration of the data derived from health determinants such as diet, gut microbiome, data from electronic health records, physical activity measures, and data collected from wearable sensors can be used to train the artificial intelligence algorithms. The outputs can be used to make targeted personalized nutrition recommendations.

The EHRs are clinical repositories wherein longitudinal patient health information generated in any healthcare delivery setting is updated in real time. The data within are comprised of physiological measure outcomes, patient's demographics, progress notes, past medical history, laboratory reports, medical prescriptions, radiology reports, and administrative information. These data differ from those in the disease registries, claims records, or prescription databases and are specifically designed for patient care, billing purposes and prove to be important pertaining to health research (19). Until 2011, the US healthcare system reached 150 exabytes of data and at this rate the healthcare big data are estimated to reach up to zetabyte (10^21^ gigabytes) (20) level. These EHR repositories are sufficiently large and can be integrated with other—omics based databases to unravel phenotypic links between the data and other genetic risk factors (21). The worldwide EHR adoption rates has increased and it is suggested that there will soon be a billion patient visits recorded yearly in the EHR systems (22) (23). However, it is important to note that the amount of nutritional information in EHRs is limited. The collection of information regarding the daily dietary intake, meals, and food content information and integration of consumer products with the massive amounts of clinical records already stored in the EHR systems could open up a new avenue for development of precision medicine and personalized nutrition pipelines. The wearable sensors integrated with the mobile technology have become increasingly popular. The real-time parameters recorded by these wearable sensors include physical activity, calories burnt and blood glucose levels that can also be leveraged to derive precise health outcomes for each individual. The derived data integrated with the EHRs could aid the process of designing automated data analytic pipeline to tailor personalized recommendation with real time input (23, 24). With an emerging need for collection of real-time data, collaboration within the healthcare firms, insurers, and hospitals has become equally crucial. In addition exposome (25) and social determinants of health could be used to assist in the personalized nutrition based recommendations (as shown in Figure 2).

#### Use of advanced artificial intelligence methods

The healthcare sector generates a large amount of data that would promote wide studies in terms of risk evaluation, disease management, and patient stratification. The major drivers of the increase in U.S. data analytics market will include the initiative to increase adoption of EHR systems, availability of healthcare information technology big data infrastructure in healthcare, and technological advancements in genome sequencing. To derive predictive insights from patient data we would need to employ data-driven modeling because systems wide advanced computational methods and data analytics platforms can help in the organization, interpretation, and pattern extraction from the health data (5, 6). These modeling technologies would include the use of AI systems, ML classifiers, and mathematical models including differential equations (DEs), rules and agent based modeling (5, 6, 18, 26–28).

With the wider application of AI methods large data repositories have ceased to be a data warehouse and have become true brains for information and knowledge extraction. Advanced ML approaches and AI are transformative technologies (29) that can be used to—(i) develop synthetic patient populations from large-scale clinical data, (ii) conduct *in silico* clinical trials to optimize clinical trial design and (iii) compare the response to various treatment options and health outcomes. The application of ML techniques and AI technologies to build *in silico* pipelines with data analytics capabilities have the potential to tailor recommendations to achieve personalized nutrition and guide the design of human studies to improve success rate. These technologies are promising in terms of investigating the linkages between nutritional regimens and modulation of whole genome-scale molecular signatures predictive of: (i) health, (ii) future health deterioration, (iii) pre-disease, and (iv) diseased states.

During the adoption of the data analytics platform as a decision support system it is important to consider not only its scientific and functional capabilities but also the management and technology issues associated with it. The compatibility of EHRs software versions with the ML and AI technology is crucial. Further, it is important to have evidence based research regarding the success of the analytics platforms. Pilot and published studies that demonstrated improved outcomes due to the use of analytics platform (similar to the reporting process on drugs and medical devices) is required. The lack of the above listed points, were some of the reasons why the MD Anderson Cancer Center's efforts to use IBM Watson cognitive computing system, in the clinical decision making process were put on hold (https://www.healthnewsreview.org/2017/02/md-anderson-cancer-centers-ibm-watson-project-fails-journalism-related/).

##### Case study

Application of advanced ML methods generated predictive insights in 10,000 virtual patients with Crohn's disease (30). Information on changes in the immunological parameters that drive response to treatment and knowledge based on experimental immunological insights were applied to create an advanced ML model. Leber et al. developed a computational modeling pipeline to use preclinical data to test and predict the efficacy of existing and novel treatments against *Clostridium difficile* infection and make predictions about clinical outcomes (31, 32). The computational pipeline included mechanistic ordinary DE based models (33) with stochastic simulations and an ensemble of advanced ML methods. Leber et al. generated replicates through stochastic simulation of the model which are similar to virtual patients from seed populations of actual patients. This approach facilitated a complete coverage of parameter and response space (31, 32) as compared to responses obtained from averaging the data. The modeling pipeline examined the dosage effects and predicted synergisms or antagonisms of combination therapies (32). Another data driven study in personalized nutrition (15) involved the metabolic phenotyping of 800 individuals and provided personalized dietary advice. This study was highly successful for clinically useful predictive modeling (15). Zeevi et al. investigated the intra and inter-individual variability in the glycemic response to standardized meals measured in an 800-participant cohort over 7 days. A ML algorithm was devised to predict the postprandial glycemic response and a combination of dietary, anthropometric, physical activity, and gut microbiota data were used as input training dataset and the output was validated from the trained ML model in an independent cohort of 100 participants. Zeevi et al. reported that different people have widely different post meal responses to the same standardized meal. Further, they provided individually tailored dietary advice to a new group of 26 individuals based on a ML derived insight or an expert opinion from clinical dietician and similar improvements in the glycemic response were reported in both the groups (15).

Another study conducted by Mathias et al. evaluated how healthy Brazilian children and teens (aged 9–13) responded inter-individually to multi-micronutrients supplement intervention (34). Mathias et al. took into account that all the individuals were genetically and environmentally unique and utilized a comprehensive approach wherein the aggregated data from all the individuals were analyzed along with the analyses of individual responses using a variation of n-of-1 trial design (34). The n-of-1 was designed such that each participant were their own control accounting for their inter-individual variability. Mathias et al. assessed the effectiveness of nutritional intervention by comparing the changes in–omics and clinical variables recorded at baseline, 6 weeks post intervention followed by a 6-week washout period. They employed an elastic net regression model and evaluated whether multiple variables such as the baseline clinical biochemistry, blood vitamin levels and dietary intakes could explain the variation in response to the intervention at each clinical endpoint (34). The results demonstrated that multi-micronutrients mediated the physiology of the systems associated with metabolism. This result was based upon the response of the total cholesterol, LDL-cholesterol, mean corpuscular volume, and circulating levels of nine vitamin metabolites to the 6-week intervention within a duration of 2 consecutive years (34).

Thus, these case studies demonstrated that ML architecture integrated with large-scale clinical data are capable of taking into account the inter-individual variability to an assigned diet and could help in moving away from the standardized approach of making general nutrition related recommendations. During a patient visit the integration of AI powered healthcare decision support systems could provide the clinicians with the EHR information, record of physical activity, microbiome information, and aid in forming tailored health recommendations. The aim of utilizing the healthcare information technology driven framework will be used to provide a predictive, personalized, and secured solution based on personal health record.

#### Integration of electronic health records with artificial intelligence-based methods and need for new biomarkers

Along with advances in ML models, deep learning has set a new trend (35) and advancements in computer vision has revolutionized clinical image analysis and widened the possibilities of complex tasks that the human brain can perform. For example, a deep convolutional neural network trained on thousands of clinical images of skin lesions outperformed dermatologists in the classification task of skin cancers (36). Similar deep neural network trained on thousands of images outperformed ophthalmologists in the detection of diabetic retinopathy (37). This demonstrates the capacity of advanced AI systems to be employed as smart clinical decision support system. Similar to the application of AI in interpretation of medical images, the AI technology can be leveraged toward tracking the nutritional contents in the daily meals (18) and the data can be connected to health outcomes measures. Food tracking is an essential component of disease management for patients with chronic metabolic disorders such as pre-diabetes, diabetes, obesity, metabolic syndrome, or for people who aim to reach their recommended body mass index. The advent of mobile applications and wearable sensors can ease the tedious process of manual data entry by capturing an image of the meal that can be used to train a ML system along with input from EHRs. The ML system could be used for further processing such as the calorie content calculation (18, 38–40) that can aid the monitoring of food intake and change in health outcomes. One such study used a ML algorithm, trained the model with a dataset of images obtained from 23 different restaurants, and accurately determined the contents and calories in a meal (40). However, it is important to note that these methods need to be fine-tuned and improved for meals that are highly complex in terms of multiple ingredients and spices (41). In healthcare one of the most important components of AI system are physicians that drive and review the predictions regarding patient healthcare. The personal health data can be used to train the AI platforms and the prediction can be reviewed by the clinicians along with patient reports to aid in the decision-making process. After the doctors review the AI recommended suggestions they can then prescribe or make alternative dietary or medication suggestions. Once the doctor determines the recommendations, the AI system can be designed to send the prescriptions directly to patient's pharmacy and notify the patients in real time regarding any abnormalities or alternative medication suggestions.

Further, modeling systems that integrate the patient characteristics from different sources of data ranging from molecular to clinical cohort scales can aid the predictive *in silico* testing of new nutritional interventions. These advanced analytics approaches can be employed to identify new molecular bio signatures as biomarkers which would be capable of defining individual responses, diversity and variation to a specific diet. The biomarker analysis tends to vary per individual especially based on age and other physiological factors. For instance, an increase in age changes the expression of gene or protein over time, for e.g., a disease biomarker for a younger adult will be different compared to an elderly. Although, there are available biomarkers for nutrition which includes the measure of essential dietary acids in plasma, protein intake, sodium and potassium levels (42), new biomarkers that would reflect the overall dietary pattern are needed. These new biomarkers and biomarker variation linked to dietary changes can be utilized as additional features for training the ML models to make accurate predictions of a personalized diet for an individual.

### Challenge 2: need for personalized nutrition computational infrastructure

In spite of nutrition research gaining enormous support due to the revolutionary -omic and data science era, there is an urgent need to deploy personalized nutrition computational infrastructure. When considering this it is important to take into account the challenges involved in the food intake databases. The food intake databases are comprised of the ability to capture complex eating patterns in an organized manner translating chemicals constituents in the food to intake of energy and nutrients. The existing tools for monitoring the food intake include the food diaries but those are challenging in terms of converting the food descriptions to the energy. An effort of moving toward an informatics infrastructure can be advantageous in terms of tailored nutrition databases that can create an environment of standard formats, annotations and network based systems to enhance food monitoring and intake processes (43). However, differences can exist in terms of food description and methods used to collect the information. These include the methods used to generate compositional values when the food is collected from different sources such as the laboratory, patient, or hospital-based sources.

Personalized nutrition and health research can benefit from building research infrastructures (44). The development of personalized nutrition user-friendly platforms can enable the interrogation of data from different resources, multiple studies, research groups at different levels comprised of molecular, cellular, tissue, organ, and population level. This personalized nutrition platform is required to take into account a systems-based approach for the identification of components involved in the human well-being and optimal health. The infrastructure build should enable the collection of information from each individual with a focus on n-of-1, as opposed to population-level data wherein an average response data limits its translatability (45). It is known that the variation in response to nutritional factors can be explained by a set of identifiable factors such as genetic, environmental, and behavioral factors that are specific to any individual (46). The advent of self-monitoring devices that facilitate real-time recording of health data can facilitate this process. Although, most of the self-monitoring devices could potentially be affected due to personal bias and do not contain the scientific rigor of n-of-1 trials, they can be improved if the individuals are well informed and are made aware of the n-of-1 trail methodologies (46). The outputs from n-of-1 studies are promising ways to advance individualized medicine and gain insights regarding the comparative treatment effect among a group of patients (47). The use of n-of-1 trails can play an important role in facilitating the process of making individualized diet recommendations as well. This would require the collection of data for one person every day or periodically over the duration of months or years. (48). The data collected from these n-of-1 trials can be mined, analyzed for trends and pattern unique to an individual (46) and help determine personal risk factors that is otherwise not possible due to the averaging of data that may come from the analysis of a group of individuals. Such inclusion of n-of-1 data in the personalized nutrition infrastructure development can benefit researchers, stakeholders, clinicians, and policy makers by providing access to individual health data and knowledge. The infrastructure can facilitate evidence based research by increasing access to the data derived from successfully implemented nutrition strategies (49). The increased access to data can provide opportunities for transdisciplinary collaboration between industrial and academic institutions and help in creating public and private partnerships.

The core requirement of the infrastructure is that it needs to be classified and identified as a food and health infrastructure (FHI). The need for research infrastructures in the specific areas of food and nutrition area were recently highlighted by the EuroDISH project (44) which mapped the existing research infrastructures and identified the gaps. The project emphasized the need for infrastructure in the **D**eterminants of food choice, **I**ntake of foods and nutrients, **S**tatus and functional markers of nutritional health and **H**ealth and disease risk (DISH model) (49). The management and implementation of the FHIs should be driven from the user level and comprise of (i) nutrition bioinformatics structures including nutrition databases for e.g., the Nutritional phenotype database (43) that is designed to facilitate the storage of biologically relevant, preprocessed –omics as well as descriptive and study participant phenotype data; (ii) data management; (iii) data processing; (iv) data sharing capabilities; and (v) platforms for publishing the data derived from the studies to a bigger community for e.g., web portals. The FHIs should aim to develop methods related to dietary assessments of the food intake and accommodate the user input regarding the daily food intake in an electronic format. The FHIs should be adaptive such that the statistical effects of the nutritional interventions can be evaluated on a continuous basis since the earlier stages.

Nutrition quality is influenced by the environment, how and where the food is grown, transported and stored (45). Additionally, the responses to diet, micro- and macronutrients differ and results from interactions of individual genetic makeup and the environment (50). These interactions between nutrition and environmental factors emphasizes the need to collect a wide variety of measures of environmental variables across the globe known alter the health outcomes. The added information about environmental variables to the FHI databases will help in understanding how the interactions between diet and human and microbial genetic diversity is affected under the environmental influence. Further, the chemical constituents in the diet are known to alter the—omics and microbial profile in humans and animal studies which emphasizes the need to include that information in the databases (45). Therefore, the establishment of a FHI will ensure that the data related to food constituents, intake, environmental variables, health determinant data, energy expenditure, and disease risk are all in one place. This FHI data can help reveal the determinants of behavior which can be utilized in the development of nutritional interventions (49). The above mentioned added measures of environmental variables and the integration of data with prior knowledge of the health relevant interactions will ultimately aid novel hypothesis generation, interpretation, and validation of results (9, 45). It is important to note that the standardization of collected data and usage of a FHI could facilitate the integration of an individual's data from other settings including the outpatient, follow-up evaluations, and discharge (51). Overall it will aid the understanding of personalized health and well-being, and predict the disease risk based on the environment, current eating habits, and health status.

The utilization of data from already existing infrastructures that can be applied and extended to the food, nutrition, and health interface is crucial. These include the application based infrastructures provided by LabKey (used to store laboratory based research projects) and REDCap (used to store patient oriented research) that can be utilized to build data linkages related to food and nutrition (5). Adherence to data standards and quality control is essential for data sharing, integration, reproducibility, and reduced query response times (5). An ideal infrastructure for personalized nutrition should standardize data formats, use standard vocabulary, and ontology. Therefore, it is crucial to develop data and software platforms that collect the food consumption data in a standardized format such as the EuroFIR (eurofir.org) (49). Further, the infrastructure should be interoperable, regularly maintained and the tools and software need to be updated with new versions. Finally, the infrastructure build should provide technical support, documentation and training to facilitate its utility.

### Challenge 3: data standardization and the requirement for training individuals

#### Standardization of the data

The use of EHRs and FHIs could ease the accessibility to patient health records that contain information determining patient care. The transition of patient records from paper to electronic format maintained in EHRs is beneficial in terms of proper diagnoses based on the patient history and cost saving thereby giving rise to better patient outcome and healthcare decisions. However, digitization in electronic format can cause—(i) improper standardization formats, (ii) erroneous documentation of diagnostic codes resulting into incorrect reporting and denial from insurance companies, (iii) lack of user engagement if not trained and additionally (iv) poorly designed technology can lead to error and give rise to issues that can cause the insufficiencies within the EHRs (52). Furthermore, the healthcare providers may report the diagnostic and billing codes inappropriately to ensure the insurance coverage for the services otherwise considered medically unnecessary by the medical insurance policy. Despite the worldwide adoption of EHR systems the processes of (i) extracting the data and transforming into standard formats, (ii) loading data from the EHRs, and (iii) reporting data to the billing department are not standardized (23, 53). It is imperative that the organization works toward—(i) standardizing the data formats, (ii) ensuring transparent communication between the physicians and billers, (iii) providing the billing staff access to provider's documentation to investigate the diagnostic codes in case of discrepancy (52). These practices would reduce the chances of medical errors and help maintain the regulatory standards. Also, the patient data sets in the records should be dynamic to facilitate the regular review and modification upon availability of additional information. Processing the data in a standard format would help the organization in the data retrieval process and is essential to streamline the process of clinical data collection to derive predictive insights. Another advantage of data standardization is that it will aid the communication across different hospitals, physicians, research institutions, and data scientists analyzing the data. The common format would ease the understanding of patient data emerging from different sources including the inpatient, outpatient or clinic and office visits for regular check-ups. Other advantages include consistent and easy identification of missing data information across different patients. Finally, data standardization across the healthcare institutions will ensure the quality of patient health care and help keep track of their records facilitating evidence based recommendations. Although, there are some initiatives including the electronic medical records and genomics that mapped phenotypic information from the EHR data to standardized formats and mapped clinical data to single nucleotide polymorphisms (54, 55).

#### Making sense of the data and training of the individuals

With the emergence of the new data driven technologies, the demand for individuals trained at the interface between computational and clinical or translational approaches has risen exponentially. Application of variety data analysis skill sets are required to interpret large and complex databases which would not only include EHRs but also external data such as claims data, public repositories, and curated data sources such as ClinVar (56) among others. In spite of the availability of open source frameworks and tools, numerous challenges are encountered in the installation, configuration, and administration of services included in the data analytics pipelines (20). The training of the individuals with integrated data science skills knowledge of biology, nutrition, biomedicine, computer science, statistics, and mathematics is required. The training curriculum for students, trainees and employees in the field of data science that is comprised of concepts from statistics, ML, bioinformatics, mathematics, and computer science needs to be regularly updated.

In 2014, under the *Modeling Immunity to Enteric Pathogens* project, a summer school and symposium on computational immunity was organized to provide experiential learning to individuals from varying backgrounds ranging from experimentalists, mathematicians, bioengineers, physicist, and nutritionists (5). The participants learned how to employ computational tools and mathematical models to deepen their analysis of the experimental data and derive new non-intuitive novel insights. Furthermore, similar regular educational boot camps, and training programs, should be organized for physicians, clinicians, nutritionists, and dieticians who will directly interact with the patients. These workshops and training session will bring researchers from a diverse background and perspectives and aid in the improvement of the predictability of the responses (28). The training of the new professionals should reflect the cutting-edge knowledge guided by the change in day to day informatics challenges (57). The training, tutorial and boot camp sessions can aid the trainers by providing them an overview of the latest technology available, data standards and the methodologies used to use the infrastructure services (49).

Although, the AI technologies are revolutionizing it is crucial to understand that it is a means to an end (or tools to facilitate and enable decision making) as opposed to a replacement for the human experts. The most important part of the system is the “user” that is required to make sense of large complex datasets. Humans and their expertise would be an integral part of the data knowledge discovery process. Thus, in order to utilize the advanced predictive capabilities of the AI system, a partnership of the experts with AI advanced capabilities is crucial. These can include experts in the fields of nutrition, bioinformatics, computer science, statistics, immunology, biochemistry, physiology, endocrinology, exercise science, and mathematics. Further, since the predictive models are trained on data that needs to be cleaned before the analysis, domain specific knowledge experts are an inevitable part of the discovery process. The AI technologies have accelerated to a point where it should be accessible to all (58). There are systems, such as the PennAI that provide a user-friendly interface ranging from uploading datasets, running ML analyses, visualizing the results in an intuitive manner and using the results to refine the knowledge derived. Thus, an availability of these interactive discovery environment can give access to ML technologies to health practitioners, healthcare providers, and researchers that can aid the users in understanding the data (58). However, it is important to note that these web tools will not replace the human interface.

### Challenge 4: data sparsity and need for improved methods

When handling clinical data pertaining to EHR, one of the main challenges is the high dimensionality and sparsity of the data. The EHR store every clinical event during patient visit or stay in the hospital. Zeevi et al. examined the fluctuation in glucose levels in response to nutrition, physical activity, and sleep; with a high-density data matrix on glucose levels that was available through continuous glucose monitoring (15). However, often times there are missing data in the EHRs, the types of data available in EHRs are heterogeneous, complex, and are in a mixed format of structured and unstructured form. The structured data includes data entry in template information such as patient's demographic information, clinical measurements, drug prescription, diagnoses whereas unstructured data included physicians handwritten notes. The reasons why the data are missing are classified into three major categories: missing completely at random, missing at random, and missing not at random (59). The missing completely at random arise when the differences between missing and observed values are negligible, this could be due missed measurements due to medical device breakdown. The missing at random arises when there is a systematic difference between the missing and observed values and the predicted value may be higher than actual measurement if the factors such as age or gender is not taken into account. The data are classified as missing not at random when the patients miss the appointment due to ill health or they expire. The duplications in the data can arise due to a patient experiencing the same event multiple times and being prescribed the same drug. Thus, multiple challenges stem not only from analyzing the EHR data but also from the sparsity and duplicated values in the available information. Dealing with the missing data is important as the missingness can lead to biased and misleading results. In cases where there are no missing values, regressions, and principal component analysis methods are used. The implementation of these methods removes variables with missing values or remove patient datasets with missing values only when the number of missing data patients is a small number. The employed estimation of missing value comprises of deterministic methods such as mean or median imputation, K-nearest neighbors (60), and stochastic methods such as the multiple imputation using chained equations process methods (60–62).

The imputation with mean and mean is easy to implement wherein the missing values are substituted with the mean or median values from the distribution of data, however, that introduces biases, and large errors if the missing value belonged to the tail of the distribution (60). In case of the K-nearest neighbors, the values of the missing data can be estimated based on the values from the individuals that are clustered together in a group (60). The values from the grouped individuals can be averaged and assigned to the missing variable. However, the K-nearest neighbors methods may fail in cases where individuals cannot be well separated in groups based on their clinical record values (60). The stochastic method including the multiple imputation by chained equations, is a framework for applying various imputation algorithms. The missing value of a variable for an individual is imputed by considering the value of other observed variables within the individual dataset, the relation between the variables and the value of the variable of interest observed in other individuals. The process is repeated for number of iterations and the imputed values are used as training set to update the estimates for second iteration (60). Lastly, it is important to remember that the stochastic processes are not free of biases as well. The assumption in multiple imputation methods is that the data are considered to be normally distributed, thus excluding non-normally distributed variables can add bias (59). Further, multiple imputation methods are computationally intensive and the algorithms require the run length proportional to the volume of missing data (59). The decision making process involved in selecting the data imputation method comprises of the data dimensionality, number of individuals, relationship among the variable, amount, and pattern of missingness time and performance of a method (60).

Since, the high dimensionality and rich volume of EHR data are valuable for personalized nutrition research it is crucial to develop improved methods to deal with the missingness. The currently available imputation methods introduce bias errors and are computational intensive. Also, the required run time for the imputation methods increases with the volume of data. Along with improved methods, better documentation along with well-versed knowledge in statistical methods regarding missing data can help.

## Concluding remarks

An individual's nutritional status can be determined by the integration of various factors including food intake, physiological health, diet, and nutrition, -omics, metabolism, and physical activity measures. To make accurate personalized nutrition recommendations and accelerate the goal of better and health well-being, advanced computational technologies such as AI, ML, and deep learning are promising in terms of providing an integrated framework. The use of data-driven methods will require the development of a personalized food and health infrastructure system comprised of advanced computational technologies with data storage, processing and sharing capabilities. The integrated and standardized infrastructure system will strengthen and enhance the patient care based on the collection of longitudinal data related to physiological measures, gut microbiome and other relevant biomarker measures. From legal and ethical consideration, it is important to take into who will have access to an individual's personal data. It is important to protect the privacy of data and prevent discrimination in terms of—eligibility for health insurance from the insurance companies, services from the hospitals and hiring decisions or terms from the employers. Overall, the standardized personalized nutrition framework approaches with protection of patient privacy can help establish preventive and predictive guidelines for promotion of health and better disease management.

## Author contributions

MV, RH, VA, and JB-R designed the architecture of the review. MV, NT-J, RH, and JB-R performed the searches for the review. MV, VA, RH, and JB-R helped with the edits. MV and JB-R wrote the manuscript.

### Conflict of interest statement

The authors declare that the research was conducted in the absence of any commercial or financial relationships that could be construed as a potential conflict of interest.

## Abbreviations

ML: Machine learning
AL: Artificial Intelligence
EHR: Electronic health record
FHI: Food health infrastructure.
